# Cellular Mechanism Underlying Hydrogen Sulfide Mediated Epithelial K^+^ Secretion in Rat Epididymis

**DOI:** 10.3389/fphys.2018.01886

**Published:** 2019-01-07

**Authors:** Dong-Dong Gao, Jia-Wen Xu, Wei-Bing Qin, Lei Peng, Zhuo-Er Qiu, Long-Long Wang, Chong-Feng Lan, Xiao-Nian Cao, Jian-Bang Xu, Yun-Xin Zhu, Yun-Ge Tang, Yi-Lin Zhang, Wen-Liang Zhou

**Affiliations:** ^1^School of Life Sciences, Sun Yat-sen University, Guangzhou, China; ^2^Key Laboratory of Male Reproductive and Genetics, National Health and Family Planning Commission, Guangzhou, China

**Keywords:** H_2_S, K^+^ secretion, epididymal epithelium, K_ATP_ channel, BK_Ca_ channel

## Abstract

As a novel gasotransmitter, hydrogen sulfide (H_2_S) elicits various physiological actions including smooth muscle relaxation and promotion of transepithelial ion transport. However, the pro-secretory function of H_2_S in the male reproductive system remains largely unclear. The aim of this study is to elucidate the possible roles of H_2_S in modulating rat epididymal intraluminal ionic microenvironment essential for sperm storage. The results revealed that endogenous H_2_S-generating enzymes cystathionine β-synthetase (CBS) and cystathionine γ-lyase (CSE) were both expressed in rat epididymis. CBS located predominantly in epithelial cells whilst CSE expressed primarily in smooth muscle cells. The relative expression level of CBS and CSE escalated from caput to cauda regions of epididymis, which was paralleled to the progressively increasing production of endogenous H_2_S. The effect of H_2_S on epididymal epithelial ion transportation was investigated using short-circuit current (*I*_SC_), measurement of intracellular ion concentration and *in vivo* rat epididymal microperfusion. Our data showed that H_2_S induced transepithelial K^+^ secretion via adenosine triphosphate-sensitive K^+^ (K_ATP_) channel and large conductance Ca^2+^-activated K^+^ (BK_Ca_) channel. Transient receptor potential vanilloid 4 (TRPV4) channel-mediated Ca^2+^ influx was implicated in the activation of BK_Ca_ channel. *In vivo* studies further demonstrated that H_2_S promoted K^+^ secretion in rat epididymal epithelium. Inhibition of endogenous H_2_S synthesis caused a significant decrease in K^+^ concentration of cauda epididymal intraluminal fluid. Moreover, our data demonstrated that high extracellular K^+^ concentration actively depressed the motility of cauda epididymal sperm in a pH-independent manner. Collectively, the present study demonstrated that H_2_S was vital to the formation of high K^+^ concentration in epididymal intraluminal fluid by promoting the transepithelial K^+^ secretion, which might contribute to the maintenance of the cauda epididymal sperm in quiescent dormant state before ejaculation.

## Introduction

Epididymis, a well-organized mass of compactly coiled tubes, acts as the last programmed differentiation reservoir for male gametes in mammals (Cornwall, 2009; Bedford, 2015; Sullivan and Mieusset, 2016). During transit through the epididymal tubule, sperm sequentially acquire their fertilizing capacity and forward motility relying on the appropriate epididymal intraluminal microenvironment (Hinton and Palladino, 1995; Dacheux and Dacheux, 2014; Sullivan and Mieusset, 2016). Ultimately, the functionally matured sperm would be stored in the cauda epididymis before ejaculation and kept immobile to preserve their viability during this period (Wong and Lee, 1983; Jones and Murdoch, 1996).

The epididymis, divided into caput, corpus and cauda regions, has been suggested to be functional highly regionalized in mammals including mouse, rat, and human (Jelinsky et al., 2007; Dacheux et al., 2016). Under the elaborate regulation of secretion and reabsorption by epididymal epithelium regionally, a specific and continuously modified ionic milieu distributes along the epididymal tubule. Previous evidence has shown that the concentration of K^+^ in the intraluminal fluid gradually increase from caput to cauda regions of rat epididymis (Hinton and Palladino, 1995). Furthermore, it has been reported that trasepithelial K^+^ secretion contribute to the formation of the high K^+^ concentration microenvironment of epididymal intraluminal fluid, although the underlying mechanisms are still unclear (Levine and Marsh, 1971; Turner et al., 1977; Wong and Yeung, 1978). Over the past few decades, several K^+^ channels have been identified in epididymal epithelium of various species including the adenosine triphosphate-sensitive K^+^ channel (K_ATP_) channel (Lybaert et al., 2008), the Ca^2+^-activated K^+^ channel (K_Ca_ channel) (Huang et al., 1999) and an outwardly rectifying K^+^ channel (Chan et al., 1995). The existence of these K^+^ channels suggests that epididymal epithelial cells may play a vital role in maintaining the high K^+^ level along the epididymal intraluminal fluid.

Recently, the scientific interest in the pro-secretory function of the endogenous gasotransmitter hydrogen sulfide (H_2_S) has blossomed (Schicho et al., 2006; Ise et al., 2011; Pouokam and Diener, 2012; Takeuchi et al., 2015; Pouokam and Althaus, 2016; Sun et al., 2016). H_2_S is endogenously produced as a result of L-cysteine (L-Cys) metabolism catalyzed by cystathionine β-synthetase (CBS) and cystathionine γ-lyase (CSE) (Szabo, 2007; Olas, 2015). In the male reproductive system, the functional expression of both CBS and CSE has been identified in testis, vas deferens, prostate and corpora cavernosa. Furthermore, K_ATP_ channel (d’Emmanuele di Villa Bianca et al., 2009) and large conductance K_Ca_ channel (BK_Ca_) channel (Li et al., 2012) were suggested to be the target of endogenous H_2_S. All these hints prompted us that H_2_S might be involved in the transepithelial K^+^ secretion in rat epididymal epithelium, leading to the formation of high K^+^ concentration microenvironment essential for sperm storage in the cauda epididymis.

The present study, therefore, aimed to investigate the regulatory functions of H_2_S on rat epididymal intraluminal fluid microenvironment and uncover the possible underlying cellular mechanisms.

## Materials and Methods

### Animals

Male *Sprague-Dawley* rats were purchased from the Animal Center of Sun Yat-sen University. According to the guidelines of the Sun Yat-sen University Animal Use Committee, animals were allowed food and water *ad libitum* and housed in an appropriate circumstance with the constant room temperature (20°C) and a 12L:12D photoperiod prior to the experiments. The animal experiment in this study was carried out in accordance with the recommendations of the Guideline for ethical review of animal welfare, Standardization Administration of the P.R.C. All procedures were subject to approval by the Animal Ethical and Welfare Committee of the Institutional Animal Care and Use Committee, Sun Yat-sen University (Approval No: IACUC-DD-18-0202).

### Drugs and Chemicals

Minimum essential medium (MEM), fetal bovine serum (FBS), penicillin/streptomycin, Hanks Balanced Salt Solution, sodium pyruvate and trypsin were purchased from Gibco (Carlsbad, CA, United States). 5-Alpha-dihydrotestosterone (5α-DHT), collagenase IA, pyridoxal 5-phosphate, *O*-(carboxymethyl) hydroxylamine hemihydrochloride chloride (AOAA), DL-propargylglycine (PAG), sodium hydrosulfide hydrate (NaHS × H_2_O), sodium lactate, L-cysteine (L-Cys), bumetanide, 4-(2-hydroxyethyl)-1-piperazineethanesulfonic acid (HEPES), glibenclamide (Glib), Iberiotoxin (IbTx) and tetraethylammonium chloride (TEA) were purchased from Sigma-Aldrich (St. Louis, MO, United States). HC067047 was purchased from Tocris (Bristol, United Kingdom). NaCl, KCl, MgSO_4_, FeCl_3_, HCl, BaCl_2_, NaHCO_3_, KH_2_PO_4_, CaCl_2_, glucose, mannitol, lactic acid, normal saline, *N,N*-dimethyl-*p*-phenylenediamine sulfate, trichloroacetic acid, and zinc acetate were purchased from Guangzhou Chemical Pharmaceutical Factory (Guangzhou, China). Universal two-step detection kit (PV-9000) and DAB detection kit were purchased from ZSBIO (Beijing, China). Fluo-3 AM was purchased from Molecular Probes (Eugene, OR, United States). PBFI AM was purchased from Cayman Chemical (Ann Arbor, MI, United States).

### Solutions

Potassium phosphate buffer (pH 8.0) contained 47 mM K_2_HPO_4_ and 3 mM KH_2_PO_4_ and then dilute to 1000 ml with ultrapure water. Krebs–Henseleit (K-H) solution contained 117 mM NaCl, 4.7 mM KCl, 2.5 mM CaCl_2_, 1.2 mM MgCl_2_, 24.8 mM NaHCO_3_, 1.2 mM KH_2_PO_4_, and 11.1 mM glucose. The solution was gassed with 95% O_2_/5% CO_2_ at 32°C to attain a pH of 7.4. Normal physiological saline solution (N-PSS) contained 137 mM NaCl, 5 mM KCl, 1 mM MgCl_2_, 2.5 mM CaCl_2_, 10 mM HEPES and 10 mM glucose (pH 7.3), and the Ca^2+^-free physiological saline solution (Ca^2+^-free PSS) was prepared by omitting Ca^2+^ and adding 2 mM EGTA to the solution. 45 mM K^+^ buffer solution contained 95 mM NaCl, 45 mM KCl, 2 mM CaCl_2_, 1 mM MgSO_4_.7H_2_O, 20 mM HEPES, 1 mM sodium pyruvate, 10 mM lactic acid, 5 mM glucose and 3% (w/v) BSA. 5 mM K^+^ buffer solution contained 95 mM NaCl, 5 mM KCl, 2 mM CaCl_2_, 1 mM MgSO_4_.7H_2_O, 20 mM HEPES, 1 mM sodium pyruvate, 10 mM lactic acid, 5 mM glucose, 3% (w/v) BSA and the mannitol was employed to adjust the osmotic pressure to the level of 45 mM K^+^ bath solution. The pH was adjusted to 6.50 or 7.40 by NaOH.

### Real-Time Quantitative PCR (qPCR)

Total RNA of rat caput, corpus and cauda epididymal tissues was extracted using RNAprep pure Tissue Kit (TIANGEN BIOTECH, Beijing, China). Reverse transcription was performed according to the protocol of the PrimeScript^TM^ RT reagent Kit (Takara, Tokyo, Japan). qPCR was performed according to the manufacturer protocols of SYBR Green I testing system (TOYOBO, Osaka, Japan) on a LightCycler 480 instrument (Roche, Basel, Switzerland). Specific primer sequences were as follow: CBS forward primer, 5′-TGAGCAGATCCAATACCGCAA-3′, CBS reverse primer, 5′-ACTCTATTTCCGGGTCTGCTC-3′; CSE forward primer, 5′-TTCCAGCACTTTGCCACTCA-3′, CSE reverse primer, 5′-CGAAGGTCAAACCGAGGACT-3′; glyceraldehyde 3-phosphate dehydrogenase (GAPDH) forward primer, 5′-GGAGTCAACGGATTTGGTCGTA-3′, GAPDH reverse primer, 5′-CTTGATTTTGGAGGGATCTCGC-3′. The PCR conditions consisted of 40 cycles of denaturation at 95°C for 5 s, annealing at 58°C for 10 s, and polymerization at 72°C for 30 s. The relative quantities of mRNAs were normalized using GAPDH as the internal control gene. The amplification efficiency of CBS/CSE primer is consistent with the efficiency of GAPDH primer and 2^-ΔΔCT^ method is used for the data analysis.

### Western Blot Analysis

Total protein extract was obtained from rat caput, corpus, and cauda epididymal tissue. The equal amount of protein loaded in each lane was resolved by SDS-polyacrylamide gel and transferred onto a PVDF membrane. Membranes were blocked by 5% (w/v) BSA for 1 h at room temperature, and then incubated with mouse monoclonal antibody against CBS (1:1000; clone 3E1; Abnova, Taipei, Taiwan), CSE (1:1000; clone 4E1-1B7; Abnova, Taipei, Taiwan) overnight at 4°C. Membranes were incubated with horseradish peroxidase (HRP)-conjugated second antibody (EarthOx, Millbrae, CA, United States) diluted at 1:20000 for 1 h at room temperature. The labeled proteins were visualized using the HRP substrate kit (Tanon, Shanghai, China).

### Immunohistochemical Experiment

The standard immunohistochemical method was used to label the paraffin sections (2 μm) of rat caput, corpus and cauda epididymal tissue as described previously (Sun et al., 2016). The sections were incubated with mouse monoclonal antibody against CBS (1:100; clone 3E1; Abnova) or CSE (1:100; clone 4E1-1B7; Abnova), respectively. Meanwhile, negative controls were obtained by incubation with PBS. The following steps were performed according to the protocol of the universal two-step HRP detection system (ZSBIO, Beijing, China).

### Measurement of H_2_S Synthesis

The biosynthesis of H_2_S in rat epididymal tissue homogenates was quantitatively measured with a modified procedure as described previously (Sun et al., 2016). Briefly, fresh epididymal tissues isolated from the rat were homogenized with potassium phosphate buffer followed by centrifugation at 4°C with 4500 × *g* for 20 min to harvest the supernatant. Before the addition of L-Cys and pyridoxal 5-phosphate, the supernatant was preincubated at 32°C with or without inhibitors for 10 min and then another 10 min was needed to cool the system on ice. Absorbance at 670 nm was measured with a microplate reader. The H_2_S concentration of each sample was calculated against a calibration curve conducted by using a series of sodium hydrosulfide (NaHS) with defined concentration. The concentration of soluble protein in the supernatant of tissue homogenates was determined using the bicinchoninic acid protein assay kit (CWBIO, Beijing, China).

### Cell Culture of Rat Cauda Epididymal Epithelium

The procedure of cauda epididymal epithelium culture has been described previously (Du et al., 2006). In short, male *Sprague-Dawley* rats weighing 100–120 g were sacrificed by CO_2_ asphyxiation. After finely minced with scissors, the cauda epididymal tissue homogenate was treated successively with 0.25% (w/v) trypsin and 0.1% (w/v) collagenase IA. Then disaggregated cells were suspended in MEM completed with sodium pyruvate (1 mM), 5a-DHT (1 nM), 10% FBS, penicillin (100 IU/ml), and streptomycin (100 IU/ml). After 4–6 h, the non-epithelial cells adhered to the wall of the culture flask and the epithelial cells were seeded onto Millipore filters (0.45 cm^2^) floating on MEM completed with other supplements. These cells then were incubated at 32°C with 5% CO_2_ for 4 days before the monolayers reached confluence and were ready for the measurement of short-circuit current (*I*_SC_).

### Measurement of *I*_SC_

Primary cultured cauda epididymal epithelial confluent monolayer was clamped vertically between the two halves of an Ussing chamber, and *I*_SC_ measurement was performed as described previously (Du et al., 2006). In brief, the epididymal epithelial confluent monolayer was short-circuited using a voltage-clamp amplifier (VCC MC6, Physiologic Instruments, San Diego, CA, United States). The signal collection and analysis system (BL-420E+, Chengdu Technology & Market, Chengdu, China) was used to obtain the *I*_SC_ data. Transepithelial resistance was obtained from the Ohm law and the change of *I*_SC_ was defined as the altered *I*_SC_ value which was normalized to current change per unit area of the epithelium (ΔμA/cm^2^). The *I*_SC_ response is expressed as downward when the cation flow from the basal to the apical side of the epithelia. The value of the transient decline phase was measured at the nadir within 300 s after the application of L-Cys or NaHS and the subsequent plateau was measured for the quantification of the long-term maintenance.

### Measurement of Intracellular K^+^

The K^+^-sensitive dye PBFI AM was employed to detect the intracellular K^+^ concentration as described previously (Kasner and Ganz, 1992). Briefly, primary cultured rat epididymal epithelial cells on cover-slips were washed with N-PSS and then incubated with PBFI AM (10 μM) for 60 min at 32°C. The ratio of the fluorescences, obtained by exciting the cells with the wavelengths (340 nm/380 nm) while measuring at the emission of 500 nm through an imaging system (Olympus, IX83, Tokyo, Japan), was positively related to the intracellular K^+^ concentration. The change of the fluorescences ratio (340 nm/380 nm) after drug treatment was normalized to the initial fluorescences ratio.

### Microperfusion of Rat Cauda Epididymis

Microperfusion of rat cauda epididymis was performed as previously described (Wong and Yeung, 1978; Gao et al., 2016), with a few modifications. Adult male *Sprague-Dawley* rats weighing 400–450 g were anesthetized with 10% chloral hydrate (200 μl/100 g of body weight) through intraperitoneal injection. During the process of the experiment, appropriate doses of 10% chloral hydrate were given to maintain the animals under anesthesia. Cauda epididymis from both sides of the animal was cannulated with suitable catheters and perfused simultaneously at a rate of 10 μl/min with a perfusion solution (N-PSS) using an infusion pump (LongerPump, Baoding, China) to displace the spermatozoa and epididymal fluid (for 30 min). Theperfusate was collected in turn to a 1.5-ml Eppendorf tube through the vas deferens inserted with a polyethylene tubing (for 60 min). The applicated concentration of NaHS was 120 μM, Glib was 1 μM and IbTx was 100 nM. 50 μl of the collected samples were then diluted at a 1:100 ratio with ultrapure water, and stored at 4°C until used for the measurement of the K^+^ concentration.

### Ionic Concentration Measurement

The samples were filtered through a 0.22 μm pore filter. The concentration of K^+^ was analyzed by ion chromatography (ICS-900, Dionex, Sunnyvale, CA, United States).

### Measurement of Intracellular Ca^2+^

Before intracellular Ca^2+^ concentration measurements, epididymal epithelial cells on cover-slips were washed with N-PSS or Ca^2+^-free PSS and incubated with 10 μM fluo-3 AM for 40 min at 32°C. Cover-slips were then transferred to a 2 ml chamber perfused with N-PSS or Ca^2+^-free PSS and the fluorescence signal was recorded using a laser scanning confocal imaging system (TCS SP2, Leica Microsystems, Mannheim, Germany). The change of fluorescence intensity after drug treatment was normalized to the initial intensity.

### Disturbance of H_2_S Generation in Rat Cauda Epididymis

The intra-epididymal injection was performed according to the method described previously (Xu et al., 1985). Male Sprague-Dawley rats (300–500 g) were used in the *in vivo* study. To narcotized the animals, rats were injected with 10% chloral hydrate (200 μl/100 g of body weight) through intraperitoneal injection. To disturb the generation of endogenous H_2_S, the rats were regionally injected with AOAA (1.36 μg/100 g of body weight) and PAG (8.44 μg/100 g of body weight) in the cauda region of epididymis every 5 days. To rescue the deficiency of the generation of endogenous H_2_S, the rats were regionally injected with AOAA, PAG, and NaHS (120 μM) in the cauda region of epididymis every 5 days. Rats injected with 25 μl normal saline served as negative control. Five days after the third injection, the rat was sacrificed by CO_2_ inhalation. The microsamples of cauda epididymal intraluminal fluid were collected by micropuncture as described before (Jenkins et al., 1980). After centrifuged for 40 min at 13000 × g, the supernatant was collected and diluted at a 1:500 ratio with ultrapure water. Then the samples were filtered through a 0.22 μm pore filter and stored at 4°C until they were used for the measurement of the K^+^ concentration by ion chromatography as mentioned above.

### Computer Aided Sperm Motion Analysis (CASA)

The cauda epididymal sperm were collected as described before (Vadnais et al., 2013). After collection, the sperm were incubated in different bath solution at 37°C for 10 min. The motility parameters including the percentage of motile and forward progressives from the total sperm analyzed were measured by SCA CASA system (SCA V 5.2, MICROPTIC S.L. Viladomat, Barcelona, Spain).

### Data Analysis and Statistics

The mathematical function *y* = *A*1 + ((*A*2-*A*1)/(1 + 10 ∧ ((log *EC*50-logx)^∗^H))) was employed to fit the concentration-response curve with variable hill confidence given by parameter ‘H’ through GraphPad Prism 7.0 (GraphPad Software, Inc., San Diego, CA, United States). A1 and A2 represent the value of the bottom asymptote and the top asymptote respectively. Origin Pro 8.0 (OriginLab Corporation, Northampton, MA, United States) was used for the statistical analysis. The results were presented as means ± SD. Student’s *t*-test was performed to assess the difference between two groups. For three or more groups, data were analyzed with one-way analysis-of-variance (ANOVA) and Bonferroni analysis was used for multiple comparisons. A value of *P* < 0.05 was considered to be statistically significant.

## Results

### Expression and Localization of Endogenous H_2_S-Generating Enzymes in Rat Epididymis

**FIGURE 1:**
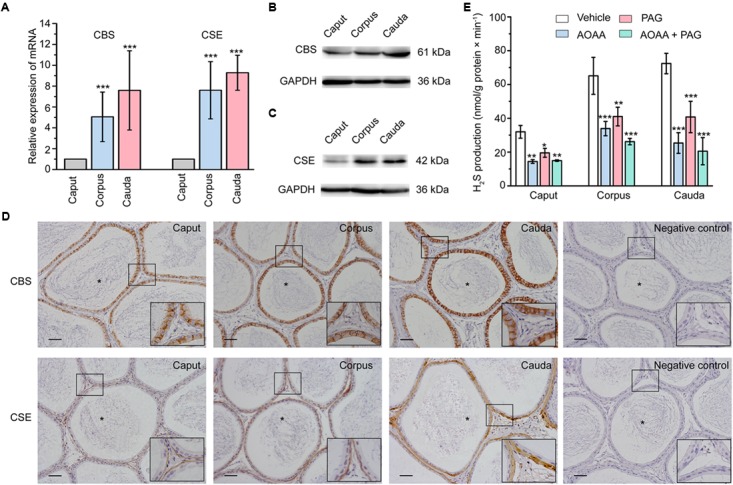
The expression, localization, and enzyme activity of CBS and CSE in rat epididymis. **(A)** Statistical analysis showing the relative mRNA level of CBS and CSE in rat caput, corpus, and cauda epididymis (*n* = 5). ^∗∗∗^*P* < 0.001 versus the corresponding caput group. Western blotting analysis showing the discriminating expression level of **(B)** CBS and **(C)** CSE protein in rat caput, corpus, and cauda epididymis. **(D)** The immunolabeling of CBS and CSE showing immunoreactivity of CBS (brown areas) was detected in the epididymal epithelium, whilst CSE (brown areas) was detected in the smooth muscle underlying the epididymal epithelium. Negative controls were prepared by substituting the primary antibody with PBS. Original magnification is 100× and 4 times magnified views of the smaller boxed areas are shown next to the lower bound. The lumen of rat epididymis is indicated by the asterisks. Scale bars: 100 μm. **(E)** Statistical analysis showing the production of H_2_S by rat caput, corpus and cauda epididymal tissue homogenate in the presence or absence of AOAA (1 mM) and/or PAG (10 mM) (*n* = 4). ^∗^*P* < 0.05, ^∗∗^*P* < 0.01, ^∗∗∗^*P* < 0.001 versus the corresponding vehicle control group. Symbols and bars indicated the means ± SD.

Using real-time quantitative PCR (qPCR), the relative mRNA level of the endogenous H_2_S-generating enzymes CBS and CSE was found to be highly expressed in corpus and cauda regions compared with caput region of the rat epididymis (Figure 1A). Consistently, the western-blot analysis showed the similar tendency of the relative protein expression level of CBS and CSE (Figures 1B,C). The cellular localization of these endogenous H_2_S-generating enzymes in rat epididymal tissue was detected by immunohistochemical analysis. As illustrated in Figure 1D, the immunolabeling of CBS was exclusively localized in the rat epididymal epithelial cells, whilst the positive labeling of CSE was observed predominantly in the thin layer of smooth muscle cells underlying the epididymal epithelium.

### Production of H_2_S in Rat Epididymis

In light of the existence of CBS and CSE in rat epididymis, H_2_S biosynthesis in epididymal tissue homogenate was then measured. As illustrated in Figure 1E, the production of H_2_S was 31.96 ± 1.88 nmol/g protein × min^-1^ (*n* = 4), 65.09 ± 5.45 nmol/g protein × min^-1^ (*n* = 4) and 72.39 ± 3.02 nmol/g protein × min^-1^ (*n* = 4) in caput, corpus and cauda epididymis, respectively. The synthesis of H_2_S was significantly suppressed when pretreated the tissue homogenate with AOAA (1 mM), the inhibitor of CBS, or/and PAG (10 mM), the inhibitor of CSE (Aydinoglu et al., 2017). These results indicated that the epididymis possessed increasing ability to generate the endogenous H_2_S from caput to cauda regions.

### Effect of H_2_S on Cauda Epididymal Epithelial Ion Transport

**FIGURE 2:**
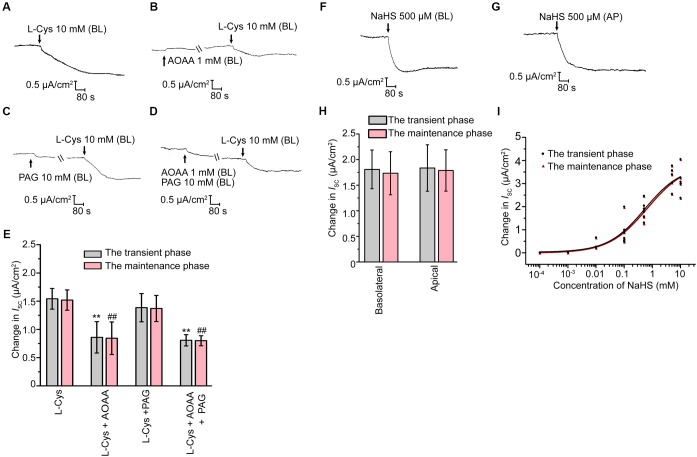
H_2_S induced *I*_SC_ response in rat cauda epididymal epithelium. **(A)** Representative trace of the *I*_SC_ responses induced by the basolateral application of L-Cys (10 mM). **(B–D)** Representative recordings of *I*_SC_ responses elicited by L-Cys (10 mM) when pretreated with AOAA (1 mM) or/and PAG (10 mM). **(E)** Statistical analysis showing the effect of the *I*_SC_ responses elicited by L-Cys (*n* = 3–5). ^∗∗^*P* < 0.01 versus the transient phase of the L-Cys group. ^##^*P* < 0.01 versus the maintenance phase of the L-Cys group. Representative trace of the *I*_SC_ responses when stimulated the confluent monolayer with **(F)** basolateral or **(G)** apical application of NaHS (500 μM). BL, basolateral application. AP, apical application. **(H)** Statistical analysis showing the effect of the *I*_SC_ responses elicited by NaHS (*n* = 6–7). **(I)** Concentration-response curve of NaHS-stimulated *I*_SC_ responses (*n* = 3–7). Symbols and bars indicated the means ± SD.

The short-circuit current (*I*_SC_) experiments were then performed to investigate the possible roles of H_2_S on rat epididymal transepithelial ion transportation. Under the unstimulated state, the primary cultured epididymal epithelial cells had a transepithelial electrical resistance of 705 ± 48 Ω × cm^2^ (*n* = 20), with a basal *I*_SC_ of 4.68 ± 0.32 μA/cm^2^ (*n* = 20) when bathed in K-H solution. Basolateral administration of L-Cys, the donor of endogenous H_2_S, induced a decrease in the *I*_SC_ response which was characterized by a transient decline phase followed by a long-term maintenance phase (Figure 2A). Interestingly, AOAA (1 mM), but not PAG (10 mM) significantly suppressed the L-Cys-stimulated *I*_SC_ response (Figures 2B–E), which was coincided with the findings that only CBS was detected in the epididymal epithelial cells. These results suggested that H_2_S might play a role in regulating the rat epididymis epithelial ion transportation. To further ascertain this hypothesis, the exogenous H_2_S donor NaHS was employed. As shown in Figures 2F–H, basolateral or apical application of NaHS induced a decrease of the *I*_SC_ response similar to L-Cys. The concentration-dependent characteristic of NaHS-stimulated *I*_SC_ response was also evaluated (Figure 2I). The half-maximal effective concentrations of NaHS were 499.7 and 600.5 μM for the transient decline phase and the long-term maintenance phase, respectively. The derived value of hill coefficient was 0.664 and 0.634 with a 95% confidence interval level. 500 μM NaHS was applied in the subsequent experiments.

### Involvement of K_ATP_ and BK_ca_ in H_2_S-Stimulated K^+^ Secretion

**FIGURE 3:**
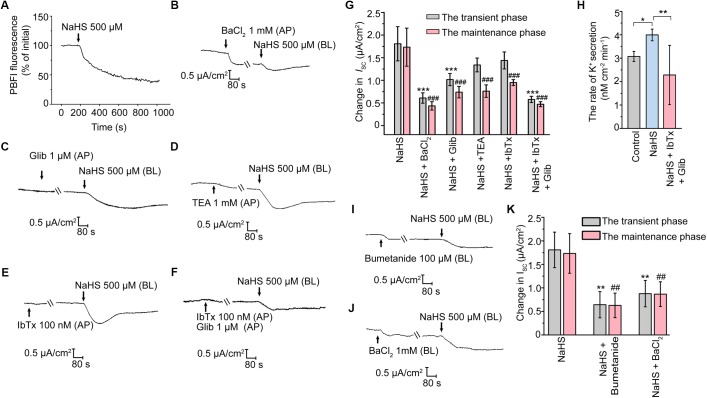
H_2_S elicited K^+^ secretion via K_ATP_ and BK_Ca_ channels in rat cauda epididymal epithelium. **(A)** Representative trace of the NaHS-induced change in intracellular K^+^ concentration. Representative trace of the NaHS-stimulated *I*_SC_ responses with apical pretreatment of **(B)** BaCl_2_ (1 mM), **(C)** Glib (1 μM), **(D)** TEA (1 mM), **(E)** IbTx (100 nM) or **(F)** both Glib (1 μM) and IbTx (100 nM). **(G)** Statistical analysis of the effect of apical K^+^ channels blockers on the NaHS-stimulated *I*_SC_ responses (*n* = 3–7). ^∗∗∗^*P* < 0.001 versus the transient phase of the NaHS group, ^###^*P* < 0.001 versus the maintenance phase of the NaHS group. **(H)** Statistical analysis showing NaHS (120 μM) promoted the rate of K^+^ secretion in rat cauda epididymis *in vivo* (*n* = 4). ^∗^*P* < 0.05, ^∗∗^*P* < 0.01 versus the control group. Representative trace of the *I*_SC_ responses stimulated by NaHS (500 μM) with the basolateral pretreatment of **(I)** bumetanide (100 μM) or **(J)** BaCl_2_ (1 mM). **(K)** Statistical analysis showing the effect of basolateral K^+^ channel blocker and NKCC inhibitor on the NaHS-stimulated *I*_SC_ responses (*n* = 5–7). ^∗∗^*P* < 0.01 versus the transient phase of the NaHS group, ^##^*P* < 0.01 versus the maintenance phase of the NaHS group. Symbols and bars indicated the means ± SD.

A decrease of *I*_SC_ response represents cation secretion or anion reabsorption. In order to investigate the possible role of H_2_S in promoting K^+^ secretion, the measurement of intracellular K^+^ concentration was performed. As illustrated in Figure 3A, the fluorescence ratio of PBFI AM (340 nm/380 nm) decreased significantly when the epithelial cells were exposed to 500 μM NaHS, indicating that H_2_S induced K^+^ secretion in rat epididymal epithelial cells. With the aim of determining the K^+^ channels involved in the H_2_S-stimulated K^+^ secretion, a series of K^+^ channels blockers were employed. When apically pretreated with BaCl_2_ (1 mM), a non-selective blocker of K^+^ channels, or Glib (1 μM), a selective blocker of K_ATP_ channel (Lin et al., 2018), the NaHS-stimulated transient decline phase and long-term maintenance phase of K^+^ secretion were both significantly suppressed (Figures 3B,C,G). However, TEA (1 mM), a non-selective blocker of K_Ca_ channels, or IbTx (100 nM), a selective blocker of BK_Ca_ channel (Li et al., 2018), significantly depressed the long-term maintenance phase of NaHS-stimulated *I*_SC_ response rather than the transient decline phase (Figures 3D,E,G). Moreover, the NaHS-stimulated *I*_SC_ response was almost abolished when pretreated with Glib (1 μM) and IbTx (100 nM) together (Figures 3F,G). Consistent with the *in vitro* findings, our *in vivo* data also manifested that H_2_S stimulated the K^+^ secretion of rat epididymal epithelium. As shown, the calculated rate of the K^+^ secretion was 3.99 ± 0.25 nmol/cm^2^/min (*n* = 4, Figure 3H) when NaHS (120 μM) was applicated, which was significantly higher than the basic rate of K^+^ secretion (3.07 ± 0.22 nmol/cm^2^/min, *n* = 4, Figure 3H) by using microperfusion of rat cauda epididymis. Likewise, the promotion of the secretion rate of K^+^ by NaHS could be significantly depressed when Glib (1 μM) and IbTx (100 nM) were applicated (2.28 ± 1.27 nmol/cm^2^/min, *n* = 4, Figure 3H). These results confirmed that H_2_S stimulated K^+^ secretion via activating K_ATP_ channel and BK_Ca_ channel in the apical epithelial cell membrane of rat epididymal epithelium.

It has long been known that basolateral Na^+^-K^+^-2Cl^-^ cotransporters (NKCC) are responsible for supplying substrate during transepithelial secretion (Bachmann et al., 2011). On the other hand, the basolateral K^+^ channels are also involved in the process of K^+^ secretion indirectly by maintaining the K^+^ circulation in the basement membrane (Sun et al., 2014, 2016). Notably, when the epididymal epithelial cells were basolaterally pretreated with bumetanide (100 μM), an inhibitor of NKCC, or BaCl_2_ (1 mM), the NaHS-stimulated *I*_SC_ response was significantly suppressed (Figures 3I–K). These observations suggested that the basolateral NKCC and K^+^ channels were implicated in H_2_S-induced transepithelial K^+^ secretion.

### Activation of BK_Ca_ Is Dependent on TRPV4

**FIGURE 4:**
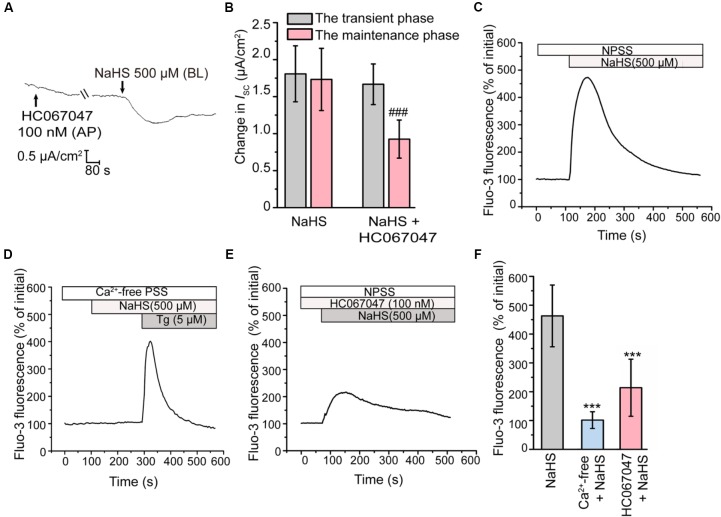
Involvement of TRPV4 in the H_2_S-induced K^+^ secretion. **(A)** Representative trace of the *I*_SC_ responses stimulated by NaHS (500 μM) with the apical pretreatment of HC067047 (100 nM). **(B)** Statistical analysis showing the effect of TRPV4 channel blocker on the NaHS-stimulated *I*_SC_ responses (*n* = 6–7). ^###^*P* < 0.001 versus the maintenance phase of the NaHS group. **(C–E)** Fluo-3 fluorescence was measured to detect the intracellular Ca^2+^ transients elicited by NaHS (500 μM) in **(C)** NPSS and **(D)** Ca^2+^-free PSS. **(E)** Representative trace of the NaHS-stimulated Ca^2+^ transients responses when pretreated with HC067047 (100 nM). **(F)** Statistical analysis showing the NaHS-stimulated Ca^2+^ transients responses in various conditions (*n* = 30–78). ^∗∗∗^*P* < 0.001 versus the NaHS group. Symbols and bars indicated the means ± SD.

Previous studies have revealed that H_2_S could induce the transient receptor potential vanilloid 4 (TRPV4) channel-dependent elevation of intracellular Ca^2+^ level and subsequently active BK_Ca_ channel in epithelial and endothelial cells (Reiter et al., 2006; Naik et al., 2016). In the present study, when apically pretreated the epithelial cells with HC067047 (100 nM), a selective blocker of TRPV4 (Everaerts et al., 2010), the long-term maintenance phase of the NaHS-stimulated K^+^ secretion was significantly suppressed (Figures 4A,B), which was consistent with that pretreated with TEA or IbTx. This result indicated that TRPV4 might be involved in the activation of BK_Ca_ channel in rat epididymal epithelium.

Subsequently, the intracellular Ca^2+^ concentration of epididymal epithelial cells was also measured. Figure 4C showed that NaHS could induce a considerable increase in intracellular Ca^2+^ concentration. However, removal of ambient Ca^2+^ abolished this response (Figures 4D,F). These observations indicated that the increase of intracellular Ca^2+^ level elicited by H_2_S was mediated by Ca^2+^ influx. Furthermore, the Ca^2+^ influx elicited by NaHS was significantly suppressed by HC067047 (100 nM) (Figures 4E,F), confirmed the involvement of TRPV4 channel in this process.

### Involvement of CBS/CSE-H_2_S Pathway in the Formation of the High K^+^ Level Fluid Environment Essential for Sperm Viability Preservation

**FIGURE 5:**
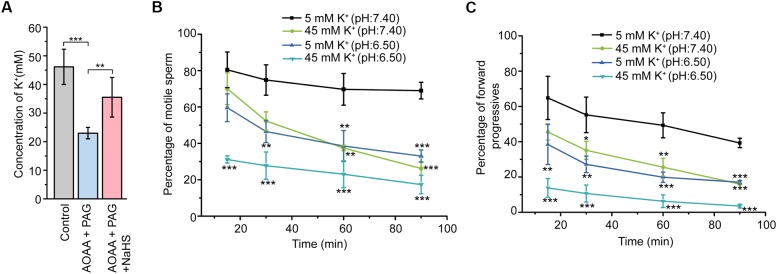
Involvement of CBS/CSE-H_2_S pathway in the formation of the high K^+^ level fluid environment essential for sperm store in the quiescent state. **(A)** Statistical analysis showing the concentration of K^+^ in the epididymal intraluminal fluid obtained from rat models (*n* = 6–8), ^∗∗^*P* < 0.01, ^∗∗∗^*P* < 0.001 versus the control group. Statistical analysis showing the percentage of motile sperm **(B)** and the percentage of forward progressives **(C)** of the epididymal sperm incubated in different bath solutions (*n* = 3). ^∗^*P* < 0.05, ^∗∗^*P* < 0.01, ^∗∗∗^*P* < 0.001 versus the 5 mM K^+^ (pH: 7.40) group. Symbols and bars indicated the means ± SD.

In view that H_2_S could induce marked K^+^ secretion of epididymal epithelium, we next sought to validate the possible roles of endogenous H_2_S in the formation of the high K^+^ concentration of the epididymal intraluminal fluid. As illustrated in Figure 5A, disturbance of the H_2_S generation in the rat cauda epididymis by local injection of AOAA (1.36 μg/100 g of body weight) and PAG (8.44 μg/100 g of body weight) significantly decreased the K^+^ concentration in rat cauda epididymal intraluminal fluid. However, when NaHS (120 μM) was supplied, the decline of the K^+^ level was significantly suppressed, indicating that endogenous H_2_S contributed to the formation of the high K^+^ level in rat cauda epididymal intraluminal fluid.

To illuminate the physiological function of the high K^+^ environment on sperm storage, the motility of cauda epididymal sperm in different conditions was evaluated by CASA. As illustrated in Figure 5B, sperm incubated in the 5 mM K^+^ buffer solution with the pH at 7.40 severed as the control group. Reduction of the pH value (from 7.40 to 6.50) or increase of the extracellular K^+^ level (from 5 to 45 mM) in the buffer solution suppressed the motility of sperm in a time-dependent manner. Interestingly, when the sperm was incubated in the 45 mM K^+^ buffer solution with the pH at 6.50, the motility of sperm was suppressed to the greatest extent within 15 min. Similar tendency was observed in another sperm motile parameter, the percentage of forward progressives (Figure 5C). These data suggested that the high extracellular K^+^ level of cauda epididymal intraluminal fluid contributed to maintaining the cauda epididymal sperm in the quiescent state in a pH-independent manner.

## Discussion

**FIGURE 6:**
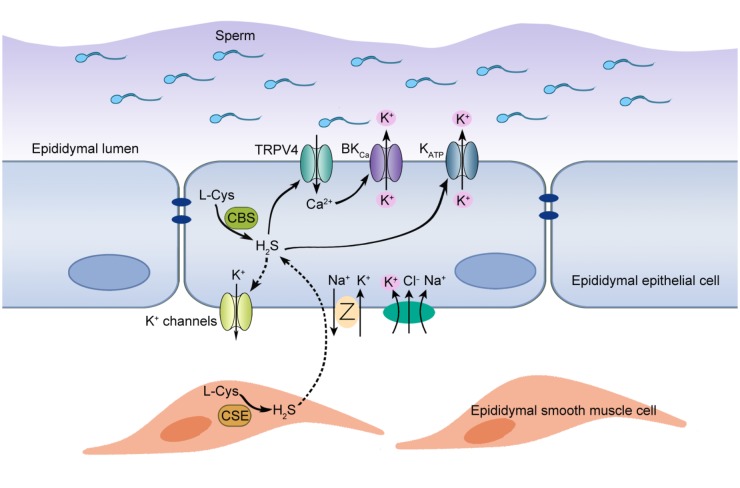
Proposed working model of H_2_S-induced K^+^ secretion in rat cauda epididymal epithelial cells. H_2_S, the enzymolysis products of CBS and CSE in the rat epididymis, induced a transepithelial K^+^ secretion mediated by K_ATP_ and BK_Ca_ channels. TRPV4 channel-mediated Ca^2+^ influx was implicated in the activation of BK_Ca_ channel. Meanwhile, basolateral NKCC and K^+^ channels participated in this process by supplying substrate K^+^ and generating driving force. Furthermore, H_2_S-stimulated K^+^ secretion was vital to maintain the high K^+^ level of intraluminal fluid, which contributed to maintaining the cauda epididymal sperm in the quiescent state in a pH-independent manner before ejaculation.

As a member of gasotransmitters, H_2_S has been identified in several tissues of male reproductive system from various mammalian species (Sugiura et al., 2005; d’Emmanuele di Villa Bianca et al., 2009; Li et al., 2011; di Villa Bianca et al., 2015). However, the existence and the functional roles of H_2_S in epididymis remain unclear. Here we have, for the first time, provided the evidence of H_2_S biosynthesis in rat epididymis and demonstrated that the pro-secretion function of H_2_S on epididymal epithelium was mediated by K_ATP_ channel and BK_Ca_ channel. Furthermore, we found that L-Cys-CBS/CSE-H_2_S pathway played an important role in the maintenance of high K^+^ concentration in rat cauda epididymal intraluminal fluid, which might contribute to the maintenance of the cauda epididymal sperm in quiescent dormant state before ejaculation. The working model to illustrate the possible role of endogenous H_2_S in rat epididymis was shown in Figure 6.

In the male reproductive system, endogenous H_2_S-generating enzymes CBS and CSE have been identified in human penile, rat testis, and vas deferens (Sugiura et al., 2005; Li et al., 2012; di Villa Bianca et al., 2015). Similar to rat testis, our results demonstrated the distribution of these two enzymes was distinct in rat epididymal tissue. CBS exclusively expressed in the epithelial cells, whilst CSE was detected predominantly in the smooth muscle cells. In consideration of the pro-secretion and relaxant function of H_2_S (Dominy and Stipanuk, 2004; Pouokam et al., 2011; Sun et al., 2016), we conjectured that CBS and CSE probably play discriminating roles in the regulation of rat epididymal transepithelial ion transportation and the rhythmic contraction, although more investigations are needed to verify this hypothesis.

Previous studies have demonstrated that H_2_S induced a biphasic change of I_SC_ response including the K^+^ secretion and the subsequent Cl^-^ secretion in vaginal epithelium (Sun et al., 2016). Furthermore, a polyphasic change in I_SC_ including a biphasic Cl^-^-dependent increase and a transient K^+^-dependent decrease in colonic epithelium was also observed (Hennig and Diener, 2009). Here in our study, both H_2_S precursor and donor induced a decrease of I_SC_ response which was characterized by a transient decline phase followed by a long-term maintenance phase of transepithelial K^+^ secretion. Notably, Cl^-^-dependent increase phase of I_SC_ was absent in our study, which might be due to the specific differences between tissues and species. Besides, secretomotor submucosal neurons were reportedly indispensable for the Cl^-^ secretion induced by H_2_S in human and guinea-pig colon (Schicho et al., 2006). In the present study, the absence of secretomotor submucosal neurons in our primary cultured monolayer of rat epididymal epithelium might lead to the discrepancy mentioned above.

For the past few years, extensive experiments have verified that H_2_S is the endogenous gaseous opener of K_ATP_ channels (Zhao et al., 2001; Cheng et al., 2004; Distrutti et al., 2006). Several previous studies have also demonstrated that H_2_S activate BK_Ca_ channel in various cell types (Li et al., 2012; Jackson-Weaver et al., 2013; Huang et al., 2014), although some other studies indicate that H_2_S inhibit the activity of BK_Ca_ channel (Li et al., 2010; Telezhkin et al., 2010). Besides, it has also been reported that phosphorylation of BK_Ca_ channel could modulate the sensitivity of BK_Ca_ channel to H_2_S (Kyle and Braun, 2014). In the present study, our results confirmed that K_ATP_ channel and BK_Ca_ channel were both involved in the H_2_S-stimulated transepithelial K^+^ secretion in rat epididymal epithelium. Nevertheless, BK_Ca_ channel primarily participated in the long-term maintenance phase of H_2_S-stimulated K^+^ secretion, which was different from K_ATP_ channel. The specific cellular mechanisms underlying this process need further exploration. It has been reported that H_2_S could directly activate K_ATP_ channel by sulfhydrating the extracellular cysteine residues of SUR subunit (Mustafa et al., 2009; Jiang et al., 2010). K_ATP_ channel subunits, Kir6.2 (KCNJ11) and SUR2 (ABCC9) have also been identified in rat epididymis principal epithelial cells (Lybaert et al., 2008). Here in our study, the immediate activation of K_ATP_ channel by H_2_S implied the direct S-sulfhydration of K_ATP_ channel protein. On the other hand, the activation of K_Ca_ channels elicited by TRPV4-dependent Ca^2+^ influx in endothelial cells has been reported (Naik et al., 2016). Moreover, it has been demonstrated that H_2_S could activate TRPV4 channel through the direct sulfudration of this channel (Paul and Snyder, 2012; Naik et al., 2016). The functional coupling of TRPV4 channels and BK_Ca_ channels in detrusor smooth muscle and human bronchial epithelial cell lines have also been observed (Fernandez-Fernandez et al., 2008; Isogai et al., 2016). In this study, our results demonstrated the blockers of BK_Ca_ channel and TRPV4 channel depressed the H_2_S-stimulated K^+^ secretion in a uniform manner, suggesting the activation of TRPV4 may be an upstream cellular event of the open of BK_Ca_ channel. Besides, the measurement of intracellular Ca^2+^ level confirmed that H_2_S induced Ca^2+^ influx via TRPV4 channel. These observations in combination indicated that TRPV4-dependent Ca^2+^ influx was implicated in the activation of BK_Ca_ channel. However, further investigation is needed to verify whether there is a signaling complex comprised of TRPV4 channel and BK_Ca_ channel in rat epididymal epithelial cells.

As is known, the physiological activities of the epididymis are highly regionalized due to the differential expression of genes (Jelinsky et al., 2007; Belleannee et al., 2012). The regional differences in gene expression level along the epididymis are indispensable for the establishment of the specific intraluminal fluid microenvironment required for sperm maturation and storage. It was noticeable that the intraluminal K^+^ level was approximately 20, 38, and 50 mM in caput, corpus and cauda epididymis respectively (Levine and Marsh, 1971; Turner et al., 1977; Jenkins et al., 1980). Interestingly, the present study demonstrated that the ability of endogenous H_2_S generation increased gradually from caput to cauda epididymis. In light of the remarkable effect of H_2_S on transepithelial K^+^ secretion in rat epididymal epithelium, we postulated that H_2_S might play crucial roles in establishing the appropriate intraluminal K^+^ microenvironment in different epididymal region. Actually, our *in vivo* animal model study demonstrated that inhibition of endogenous H_2_S biosynthesis resulted in disequilibrium of the cauda epididymal intraluminal K^+^ concentration, which might provide new insight into the pro-secretory role of endogenous H_2_S. For decades, epididymal intraluminal proteins have been well-investigated for their crucial function on the maturation of the epididymal sperm (Dacheux et al., 2003; Xie et al., 2016). However, just a few studies have focused on the physiological function of ionic environment in epididymal lumen (Dacheux and Dacheux, 2014). As is known, sperm stored in the cauda epididymis will keep quiescent before ejaculation. The immobile status is essential to preserve their viability during this period (Jones and Murdoch, 1996). The acidic pH microenvironment was reported to be a key factor to maintain the cauda epididymal sperm in the quiescent state (Nishigaki et al., 2014). Besides, the relevance of the immobile status of sperm and the high level of K^+^ in external fluid environment has also been explored although the underlying mechanism remains largely unclear (Jessee and Howards, 1976; Wong and Lee, 1983). Here in our study, we demonstrated that the high extracellular K^+^ level contributed to maintain the cauda epididymal sperm in the quiescent state in a pH-independent manner, indicating the overlooked roles of extracellular K^+^ in epididymis. At the meantime, our data suggested that CBS and CSE were indispensable for the epididymis to create an appropriate microenvironment for sperm stored in the quiescent state in cauda epididymis. However, it should be noted that a previous research showed that male mice with deletion of CSE are fertile (Yang et al., 2008). We postulated that this might be due to the compensative effect of CBS. Another study validated that CBS-knockout mice suffered from severe growth retardation and a majority of them died within 5 weeks after birth (Watanabe et al., 1995), indicating that CBS was indispensable for the survival of mice, although the fertility of CBS-knockout male mice is still elusive. Therefore, the tissue-specific double knockout of CBS and CSE in rat epididymis is needed to further evaluate their functional role in male fertility.

Collectively, this study demonstrated that H_2_S could stimulate the transepithelial K^+^ secretion of rat epididymis via K_ATP_ channel and BK_Ca_ channel. Furthermore, CBS and CSE were indispensable to maintain the high K^+^ concentration of rat epididymal intraluminal fluid essential for sperm storage via L-Cys-CBS/CSE-H_2_S pathway. The physiologic effects of H_2_S elucidated in our study may provide new insight into the treatments of asthenozoospermia and male contraceptives.

## Author Contributions

D-DG, Y-LZ, and W-LZ: conception and design of the work. D-DG, J-WX, W-BQ, LP, Z-EQ, L-LW, C-FL, X-NC, J-BX, Y-XZ, and Y-GT: acquisition, analysis, or interpretation of data. D-DG and Y-LZ: article writing with contributions from other authors. J-WX: revised the manuscript. All authors approved the final manuscript and agreed to be accountable for the work, persons designated as authors qualify for authorship, and those who qualify for authorship are listed.

## Conflict of Interest Statement

The authors declare that the research was conducted in the absence of any commercial or financial relationships that could be construed as a potential conflict of interest.
